# Haemangioma of Knee Joint: A Case Report

**DOI:** 10.5704/MOJ.1407.002

**Published:** 2014-07

**Authors:** P Choudhari, Anand Ajmera

**Affiliations:** Department of Orthopaedics & Traumatology, Sri Aurobindo Institute of Medical Sciences, Indore, India; Department of Orthopaedics & Traumatology, Sri Aurobindo Institute of Medical Sciences, Indore, India

## Abstract

*Background:*Hemagioma arising in the knee is a rare cause of knee swelling. The diagnosis frequently is delayed for long.

*Methods:*We are presenting the case report of a 12 year old male child who had a swelling on anteromedial knee which remained undiagnosed for more than 1 year. Patient had all baseline blood investigations and plain radiograph of knee which were normal. He also had got his knee aspirated which yielded only blood.

*Conclusion:*The aim of presenting this case report is to create awareness about the possibility of a hemangioma arising from a joint which although rare should be kept as a differential diagnosis.

**Key Words:**

Haemangioma,knee joint,Swelling

## Introduction

Haemagioma arising in the knee is a rare cause of knee swelling. The diagnosis frequently is delayed for long. We are presenting the case report of a 12- year boy who had a swelling on the anteromedial aspect of the left knee which remained undiagnosed for more than a year.

Patient had all baseline blood investigations and plain radiograph of knee which were normal. He Aspiration of the knee had yielded only blood. The aim of presenting this case report is to create awareness about the possibility of a haemangioma arising from a joint which although rare should be considered as a differential diagnosis.

## Case Report

Haemangiomas arising in a joint are rare. Amongst all the joints involved, the knee is the most common followed much less commonly by elbow, wrist and ankle^1^. Haemangioma of the knee can present with a visible swelling, associated with pain, and can be a cause of spontaneous haemarthrosis in children and young adults. Two different forms have been observed synovial haemangioma and arteriovenous malformation (Hemangio-hamartoma)^2^. Both these types can cause a chronic haemorrhagic synovitis with ultimate joint degeneration as they tend to remain undiagnosed for long periods.

**Fig. 1: Preoperative photograph showing the anteromedial knee swelling.**
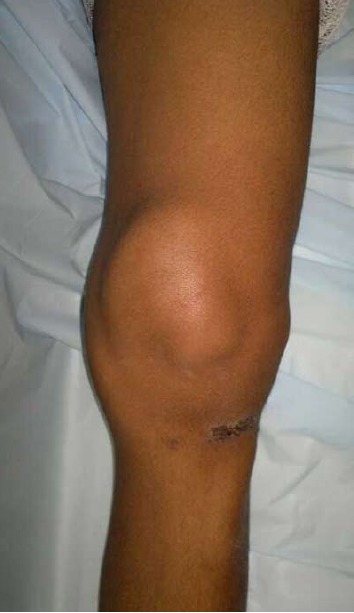


We report a case of a 12- year old boy who presented with a localized swelling over the antero-medial aspect of left knee for a year. The patient had no history of trauma or complaints in any other joint. There were no constitutional symptoms. Pain was moderate and bearable. Predominantly it was the swelling for which patient came to the hospital. On examination patient had a well-defined swelling on the superior antero-medial -aspect of right knee, measuring 10 x 7 cm x 4 cm. It was mildly tender, not attached to the deeper structures or skin, and soft in consistency. The knee had a full range of movements without any - demonstrable instability. There was no knee effusion. The swelling was more prominent with the knee flexed and less so with the knee extended. (Figure 1)

Antero-posterior and lateral views of the left knee were normal apart from the soft tissue shadow of the lesion seen on AP view. Blood counts and coagulation parameters were normal. Patient gave a history of aspiration on one occasion in another hospital which according to the records had yielded only blood.

**Fig. 2: MRI images showing the lesion between patella and femoral condyle.**
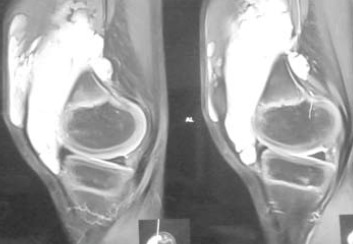


An MRI of the right knee showed a large lobulated altered intensity mass in the superomedial region of knee extending into the suprapatellar space. Multiple hypointense septae were seen on T2W images (Figure 2). The appearances were suggestive of a benign lesion, possibly a haemangioma.

**Fig. 3: Intraoperative photograph showing the Haemangioma.**
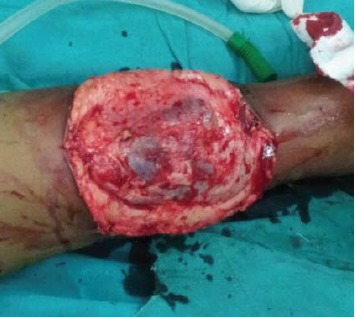


A decision was made for an excisional biopsy of the lesion under spinal anaesthesia. A thigh tourniquet was applied but not inflated. Through a n antero-medial arthrotomy of the knee the lesion was excised in toto carefully using electrocautery and meticulous haemostasis at all stages. The macroscopic appearance of the lesion was a reddish brown lobulated mass (Figure 3). Contrary to the expectation, there was not much bleeding even though the tourniquet was not inflated. A compression dressing was applied after skin closure. Histopathological examination of the excised specimen confirmed the diagnosis of a cavernous synovial haemangioma

Post operative recovery was uneventful. The knee was kept in a compression dressing for two weeks. Knee mobilization exercises were started within 48 hours of the surgery. At one month post-operative follow up the patient was doing extremely well, with a full range of left knee movement. There was no evidence of recurrence of the swelling.

## Discussion

Synovial haemangiomata are a rare cause of knee swelling and pain. Up to the present time, about 200 cases have been reported in the world literature3

As early as 1949 Julian E Jacobs et al ^4^ had stated “Articular hemangiomata can be diagnosed prior to surgery in practically all cases, provided the correlation between the clinical picture and the pathological process was fully appreciated. These signs are significant: ^1^ the presence of a circumscribed mass, which is covered by normal skin and which increases in size when the extremity is in the dependent position; ^2^ the presence of blood after puncture of the mass; and ^3^ the disappearance of the contrast substance roentgenographically after injection into the vascular area. Surgical excision offers excellent end results.”

In our patient localized swelling over the supero-medial aspect of the knee was the only presenting symptom. History of recurrent non-traumatic haaemarthrosis in a patient with a normal coagulation profile should raise a suspicion of synovial haemangioma. The X-rays are essentially normal in most of these cases and may show just a soft tissue shadow. At times calcification or phleboliths may be seen on the X-rays. Less than 5% of patients show periosteal reaction, cortical destruction, osteoporosis, advanced maturation of the epiphyses and a discrepancy in leg length or even arthropathy simulating haemophilia . Angiography as an investigation also provides the opportunity for therapeutic embolisation of a major feeder vessel in the same sitting. Angiography may fail to show the hamangioma if the vessels are thrombosed. MRI is the investigation of choice for diagnosis of synovial haemangiomata. Contrast MRI may be used to differentiate haemangioma from joint fluid in cases where there is a knee effusion.

Several treatment methods have been advocated for synovial haemangioma in the past, like radiotherapy, surgical excision (open or arthroscopic), and arthroscopic laser ablation. Arthroscopic excision is possible for pedunculated or focal lesions of small size. As the results of open excision are good it is the treatment of choice for large lesions. Synovial hemangiomata should be treated early as they can cause arthropathy, due to recurrent episodes of intra-articular bleeding, and they can even infiltrate muscles, fat and cortical bone 5.

## Conclusion

Synovial hemangioma arising in a joint is rare. Knee joint is most commonly affected. Recurrent episodes of nontraumatic haemarthrosis along with normal coagulation parameters should raise the possibility of synovial haemangioma. Plain radiographs are of limited help and MRI of the knee is the investigation of choice for confirming the diagnosis. Angiography is of value but is invasive and not available at all centers. Once the diagnosis is confirmed, early excision should be instituted to reduce the risk of arthropathy.
